# Editorial: The emerging role of protein methylation/demethylation modification in disease and homeostasis

**DOI:** 10.3389/fmolb.2025.1565598

**Published:** 2025-02-10

**Authors:** Yan-Jun Liu, Jian-Fei Lu, Xin Peng, Guan-Jun Yang

**Affiliations:** ^1^ State Key Laboratory for Quality and Safety of Agro-Products, School of Marine Sciences, Ningbo University, Ningbo, Zhejiang, China; ^2^ Ningbo Municipal Hospital of TCM, Affiliated Hospital of Zhejiang Chinese Medical University, Ningbo, Zhejiang, China

**Keywords:** protein methylation/demethylation, DNA methylation, RNA demethylation, posttranslational modifications, demethylase, methyltransferase

## Introduction

Protein methylation and demethylation are pivotal post-translational modifications that significantly influence cellular functions and processes. Methylation, catalyzed by protein methyltransferases, transfers a methyl group from S-adenosylmethionine (SAM) to target amino acids. This seemingly simple addition can have profound implications, affecting protein-protein interactions, enzymatic activity, subcellular localization, and overall protein stability. Consequently, these modifications are integral to various cellular processes, including signal transduction, gene expression, DNA repair, and protein turnover (Luo et al., 2024; Cheng et al., 2022; Yang et al., 2025). Conversely, protein demethylation, executed by protein demethylases, reverses these modifications, restoring the original state of the amino acid residues (Song et al., 2023; Li et al., 2024; Yang G. J. et al., 2021). The mechanisms of demethylation are diverse and depend on the specific amino acid and the demethylase involved. This dynamic interplay between methylation and demethylation is essential for maintaining cellular homeostasis and responding to environmental cues.

The significance of protein methylation and demethylation extends beyond basic cellular functions; they are critical in numerous biological processes, including development, differentiation, and epigenetic regulation. For instance, histone methylation is a well-studied modification that orchestrates chromatin structure and gene expression, influencing cellular identity and function. Additionally, the methylation of non-histone proteins, such as transcription factors and signaling molecules, further underscores the complexity and importance of these modifications in regulating cellular activities. These modifications are not only fundamental to basic cellular functions but also have profound implications in more complex biological phenomena and diseases. Understanding the intricate mechanisms underlying protein methylation and demethylation is crucial for unraveling their roles in normal physiology and disease pathology. As research in this area progresses, it holds the promise of revealing potential therapeutic opportunities that could transform our approach to treating various diseases.

This Research Topic collected excellent works on the “*The Emerging Role of Protein Methylation/Demethylation Modification in Disease and Homeostasis*,” and a total of four articles from 12 authors were accepted, which deepens the understanding of role of protein methylation/demethylation modification in disease and homeostasis, and highlights the clinical significance of methylation/demethylation modification in disease theranostics. This Research Topic can be roughly divided into the following three subtopics.

## DNA/RNA methylation

DNA/RNA methylation is a crucial epigenetic mechanism that plays a significant role in regulating gene expression and maintaining genome stability (Erdmann and Picard, 2020; Yang B. et al., 2021). Protocadherin gamma subfamily B, 7 (PCDHGB7) is a member of the protocadherin family, essential for neuronal connections and associated with female reproductive cancers. Yuan et al. examined PCDHGB7 expression, prognosis, biological function, methylation patterns, and its links to immune infiltration and immunotherapy response using public datasets (HPA, TCGA, GEO, OncoDB, and MEXPRESS). They also included two lung cancer immunotherapy cohorts from our clinical center to investigate the relationship between PCDHGB7 methylation and protein levels in plasma and immunotherapy outcomes. PCDHGB7 expression was found to be lower in lung adenocarcinoma (LUAD) and lung squamous cell carcinoma (LUSC), correlating with tumor prognosis. It showed a positive correlation with inhibitory immune cells and a negative correlation with tumor mutational burden (TMB) and homologous recombination deficiency (HRD). Methylation levels of PCDHGB7 were higher in tumor tissue and inversely related to PCDHGB7 mRNA levels. In immunotherapy cohorts, higher tissue expression of PCDHGB7 was associated with worse prognosis. Patients with hypermethylated PCDHGB7 in baseline plasma had shorter progression-free survival (PFS) and overall survival (OS), while those with early reductions in PCDHGB7 methylation had the best outcomes. Plasma PCDHGB7 protein levels could predict responses to immune checkpoint inhibitors and serve as a prognostic marker for PFS. Overall, PCDHGB7 expression and methylation are significant prognostic and immunological biomarkers in non-small cell lung cancer, with potential as novel predictors of immunotherapy efficacy. Meng et al. explores the hotspots and trends in RNA methylation and tumor immune cells using bibliometric analysis and visualization techniques. A thorough search of WoSCC from 2014 to 2023 identified 3,295 articles and reviews on this Research Topic. Tools like CiteSpace, Bibliometric, and VOSviewer were used for analysis and visualization. There is a notable increase in publications linking RNA methylation to tumor immune cells, with Chinese authors and institutions showing significant growth in both publication volume and influence. SUN YAT SEN UNIVERSITY leads in article output and has established extensive international collaborations, while HARVARD UNIVERSITY has also made significant contributions. Notably, Frontiers in Immunology has published the most articles in this area, and Nature Communications has released the most influential papers. The field’s ongoing vitality is supported by foundational research, including key studies by Professor Chiappinelli KB in Cell and a highly cited paper by Professor Han DL in Nature. Current research focuses include m5C, immunotherapy, and the immune microenvironment, with future studies expected to target m5C, n7-methylguanosine, cuproptosis, prognosis assessment, immunotherapeutic strategies, and the tumor microenvironment.

## RNA demethylation

RNA demethylation is the process of removing methyl groups from RNA molecules mediated by RNA demethyalses (Mathieu et al., 2023). ALKBH5 is a demethylase that regulates RNA ^m6^A modification and has recently been implicated in tissue fibrosis processes (Qu et al., 2022). Liao et al. summarized inconsistent its mechanisms and effects in fibrosis. Various cell types, including parenchymal cells, immune cells (neutrophils and T cells), macrophages, endothelial cells, and fibroblasts, contribute to different stages of fibrosis. Their review examines how ALKBH5 regulates these cells, its effects on their functions, and the resulting fibrosis outcomes. It also summarizes ALKBH5’s role in fibrotic diseases such as pulmonary, liver, cardiac, and renal fibrosis, and discusses the ALKBH5 inhibitors identified so far, highlighting its potential as a clinical target for fibrosis.

## Protein methylation

Protein methylation, catalyzed by methyltransferases, plays a crucial role in regulating various biological processes through the modification of histones and non-histone proteins (Kunadis et al., 2021; Song et al., 2016). These methyltransferases are implicated in post-translational modifications that influence gene expression and cellular signaling pathways, contributing to cancer progression and therapy resistance. Targeting their activity in a cancer-specific manner holds significant promise for reversing chemoresistance (Gillespie et al., 2024; Yamamoto et al., 2024; Li et al., 2021). Recent advances in selective inhibitors for these methyltransferases have opened new avenues for cancer treatment, particularly in overcoming drug resistance, which can arise from multiple factors. Increasing evidence highlights the role of protein methyltransferases (PMTs) in the development of chemoresistance across different cancers. PMTs, including protein lysine methyltransferases (PKMTs) and protein arginine methyltransferases (PRMTs), methylate amino acids like lysine and arginine. Many PMTs are dysregulated in cancer, yet their specific roles in chemoresistance remain poorly understood, underscoring the need for innovative methods to characterize PMTs and identify potential clinical inhibitors. Some PMTs have already led to the development of small molecule inhibitors currently being tested in clinical trials as anticancer drugs. Micallef et al. discuss the various families of dysregulated PKMTs and PRMTs in cancers, the proteins they target, and their involvement in chemoresistance, along with their inhibitors and mechanisms of action. The review also addresses current challenges and future directions for PMT inhibitors in combating cancer chemoresistance.

## References

[B1] Cheng S. Yang G. J. Wang W. Song Y. Q. Ko C. N. Han Q. B. (2022). Identification of a cytisine-based EED-EZH2 protein-protein interaction inhibitor preventing metastasis in triple-negative breast cancer cells. Acta Mater. Medica 1 (2), 197–211. 10.15212/amm-2022-0006

[B2] Erdmann R. M. Picard C. L. (2020). RNA-Directed DNA methylation. PLoS Genet. 16, e1009034. 10.1371/journal.pgen.1009034 33031395 PMC7544125

[B3] Gillespie M. Chiang K. Regan-Mochrie G. Choi S. Ward C. Sahay D. (2024). PRMT5-regulated splicing of DNA repair genes drives chemoresistance in breast cancer stem cells. Oncogene. 10.1038/s41388-024-03264-1 PMC11932929 39695328

[B4] Kunadis E. Lakiotaki E. Korkolopoulou P. Piperi C. (2021). Targeting post-translational histone modifying enzymes in glioblastoma. Pharmacol. Ther. 220, 107721. 10.1016/j.pharmthera.2020.107721 33144118

[B5] Li C. Y. Wang W. H. Leung C. H. Yang G. J. Chen J. (2024). KDM5 family as therapeutic targets in breast cancer: pathogenesis and therapeutic opportunities and challenges. Mol. Cancer 23, 109. 10.1186/s12943-024-02011-0 38769556 PMC11103982

[B6] Li X. Gera L. Zhang S. Chen Y. Lou L. Wilson L. (2021). Pharmacological inhibition of noncanonical EED-EZH2 signaling overcomes chemoresistance in prostate cancer. Theranostics 11 (14), 6873–6890. 10.7150/thno.49235 34093859 PMC8171087

[B7] Luo Y. Lu J. Lei Z. Zhu H. Rao D. Wang T. (2024). Lysine methylation modifications in tumor immunomodulation and immunotherapy: regulatory mechanisms and perspectives. Biomark. Res. 12 (1), 74. 10.1186/s40364-024-00621-w 39080807 PMC11289998

[B8] Mathieu N. F. Matthew T. Mjarb K. D. (2023). The proteins of mRNA modification: writers, readers, and erasers. Annu. Rev. Biochem. 92, 145–173. 10.1146/annurev-biochem-052521-035330 37068770 PMC10443600

[B9] Qu J. W. Yan H. M. Hou Y. F. Wen C. Liu Y. Zhang E. (2022). RNA demethylase ALKBH5 in cancer: from mechanisms to therapeutic potential. J. Hematol. Oncol. 15 (1), 8. 10.1186/s13045-022-01224-4 35063010 PMC8780705

[B10] Song Y. Wu F. Wu J. J. J. (2016). Targeting histone methylation for cancer therapy: enzymes, inhibitors, biological activity and perspectives. J. Hematol. Oncol. 9 (1), 49. 10.1186/s13045-016-0279-9 27316347 PMC4912745

[B11] Song Y. Q. Yang G. J. Ma D. L. Wang W. H. Leung C. H. (2023). The role and prospect of lysine-specific demethylases in cancer chemoresistance. Med. Res. Rev. 43, 1438–1469. 10.1002/med.21955 37012609

[B12] Yamamoto T. Hayashida T. Masugi Y. Oshikawa K. Hayakawa N. Itoh M. (2024). PRMT1 sustains *de novo* fatty acid synthesis by methylating PHGDH to drive chemoresistance in triple-negative breast cancer. Cancer Res. 84 (7), 1065–1083. 10.1158/0008-5472.CAN-23-2266 38383964 PMC10982647

[B13] Yang B. Wang J. Q. Tan Y. Yuan R. Chen Z. S. Zou C. (2021). RNA methylation and cancer treatment. Pharmacol. Res. 174, 105937. 10.1016/j.phrs.2021.105937 34648969

[B14] Yang G. J. Liu Y. J. Chen R. Y. Shi J. J. Li C. Y. Wang R. (2025). PRMT7 in cancer: structure, effects, and therapeutic potentials. Eur. J. Med. Chem. 283, 117103. 10.1016/j.ejmech.2024.117103 39615371

[B15] Yang G. J. Zhu M. H. Lu X. J. Liu Y. J. Lu J. F. Leung C. H. (2021). The emerging role of KDM5A in human cancer. J. Hematol. Oncol. 14 (1), 30. 10.1186/s13045-021-01041-1 33596982 PMC7888121

